# Retraction Note: The role of apatinib combined with paclitaxel (aluminum binding type) in platinum-resistant ovarian cancer

**DOI:** 10.1186/s13048-021-00785-1

**Published:** 2021-02-19

**Authors:** Hong Zhao, Rong Li, Xiaoyan Wang, Xin Lu, Min Hu, Jinbin Zhang, Xia Zhao, Xiaoqin Song, Yangyang Liu

**Affiliations:** 1 Department of Biochemistry and Molecular Biology, Basic Medical College, Shanxi Medical University, No. 56 Xinjian South Road, Yingze District, Taiyuan City, Shanxi Province China; 2 Department of Gynecology, Shanxi Cancer Hospital, Taiyuan, China; 3 Shanxi Province Center for Disease Control and Prevention, Taiyuan, China; 4 The Second Affiliated Hospital of Shanxi Medical University, Taiyuan, China

**Retraction Note: J Ovarian Res 13, 113 (2020)**

**https://doi.org/10.1186/s13048-020-00719-3**

The Editors-in-Chief have retracted this article [1]. Concerns were raised with Figures 2 and 3, specifically:

- Figure 2E: the panels for Group-2 and Group-3 appear to partially overlap.
- Figure 2E: the panels for nab-P and AP appear to partially overlap.
- Figure 3E: the panels for Bax/Group-1 and Bax/Group-3 appear to partially overlap.
- Figure 3E: the panels for Bax/Group-2 and bcl-2/Control group appear to partially overlap.
- Figure 3E: the panel for CD31/Group-1 appears to partially overlap with the panels for CD31/Group-3 and MMP-2/Group-2.
- Figure 3E: the panel for CD31/Group-3 appears to partially overlap with the panels for MMP-2/Group-1 and MMP-2/Group-2.
- Figure 3E: the panel for CD31/Group-3 appears to partially overlap with the panel for MMP-2/Group-1.
- Figure 3E the panels for MMP-2/Group-1 and MMP-2/Group-2 appear to partially overlap.

The Editors-in-Chief have therefore concluded that the data reported in this article are unreliable. The authors have not responded to any correspondence regarding this retraction
